# Cytomegalovirus in HIV: A Modifiable Driver of Inflammation, Frailty, and Aging

**DOI:** 10.1007/s11904-026-00775-2

**Published:** 2026-03-20

**Authors:** Alfonso Cabello Ubeda, Kristine M. Erlandson, Michael L. Freeman, Scott L. Letendre, Celestine N. Wanjalla, John R. Koethe, Michael J. Corley, Peter W. Hunt, Sara Gianella

**Affiliations:** 1 Division of Infectious Diseases, Health Research Institute - Fundación Jiménez Díaz University Hospital, Autónoma de Madrid University (IIS-FJD, UAM), Madrid, Spain; 2 Division of Infectious Diseases, Department of Medicine, University of Colorado Anschutz Medical Campus, Aurora, CO USA; 3 Division of Infectious Diseases and HIV Medicine, Department of Medicine, Case Western Reserve University, Cleveland, OH USA; 4 Division of Infectious Diseases and Global Public Health, Department of Medicine, University of California San Diego, La Jolla, CA USA; 5 Division of Infectious Diseases, Department of Medicine, Vanderbilt University Medical Center, Nashville, TN USA; 6 Division of Geriatrics, Gerontology & Palliatie Care, Department of Medicine, University of California San Diego, La Jolla, CA USA; 7 Division of Experimental Medicine, Department of Medicine, University of California San Francisco, San Francisco, CA USA

**Keywords:** Cytomegalovirus, HIV, Inflammaging, Frailty, Letermovir, Epigenetics, Adipose tissue, Cardiometabolic disease

## Abstract

**Purpose:**

Cytomegalovirus (CMV) infection is nearly universal among people with HIV (PWH) and contributes to chronic inflammation, immune senescence, and accelerated biological aging despite suppressive antiretroviral therapy (ART). This review summarizes recent advances in understanding the role of CMV in multimorbidity and aging in PWH, focusing on immune and tissue-based mechanisms, comorbidities, and emerging interventions.

**Recent Findings:**

CMV reactivation drives clonal T-cell expansion, innate immune reprogramming, adipose tissue inflammation, metabolic rewiring, and durable cellular epigenetic changes that amplify risks for vascular disease, frailty, brain health disorders, diabetes mellitus, and cancer. Early interventional data indicate that letermovir can reduce inflammation and improve immune and frailty outcomes in PWH, while vaccines are advancing in clinical evaluation.

**Summary:**

CMV is a modifiable driver of immune dysfunction and aging in PWH. Targeted antiviral, vaccine, and host-directed approaches may reduce multimorbidity and promote healthy aging, particularly in populations at greatest risk.

## Key Points

Cytomegalovirus (CMV) is nearly universal among people with HIV (PWH) and persists despite suppressive antiretroviral therapy (ART), driving chronic immune activation and accelerated biological aging.

CMV is mechanistically linked to clonal T-cell expansion, innate immune reprogramming, metabolic rewiring, and durable epigenetic imprinting.

Clinical consequences span multiple aging-related disorders, including frailty cardiovascular disease (CVD), cognitive decline, diabetes mellitus, and cancer.

Therapeutic strategies under evaluation include anti-CMV drugs like letermovir and vaccines.

Future directions emphasize precision, multimorbidity-focused interventions to extend healthspan in aging PWH.

## Introduction

 Antiretroviral therapy (ART) has transformed HIV into a chronic, manageable condition and extended the survival of people with HIV (PWH) by decades [1–3]. Yet, persistent immune activation and inflammation remain, accelerating biological aging and elevating the risk of non-AIDS comorbidities such as cardiovascular disease (CVD), frailty, cancers, and brain health disorders [4–6]. Cytomegalovirus (CMV), a ubiquitous β-herpesvirus, is highly prevalent among PWH and is a major driver of this chronic inflammatory state [7–10]. After primary exposure, CMV establishes lifelong latency with episodic reactivation, often localized to mucosal compartments. While typically asymptomatic, these episodes fuel immune dysfunction and interact with HIV to sustain reservoir persistence and multimorbidity [8, 11–14].

This review summarizes current evidence on (i) CMV epidemiology in PWH (ii), interactions between CMV and HIV on the immune system (iii), CMV-associated comorbidities, and (iv) emerging therapeutic and host-directed interventions that might improve healthy aging in PWH.

## Epidemiology of CMV

CMV is one of the most common herpesviruses, with seroprevalence ranging from ~ 50–70% in high-resource settings to > 90% in resource-limited regions [15–19]. In resource-limited settings, overcrowding and poverty facilitate early-life acquisition, commonly through vertical transmission, breastfeeding, or contact with caregivers’ saliva [20]. In high-resource regions, acquisition typically occurs during adolescence or early adulthood, with prevalence rising steadily with age [21]. Once acquired, CMV persists latently in hematopoietic stem cells and myeloid progenitors with the potential for periodic reactivation or low-level replication, particularly in the context of immunosuppression or chronic inflammation [22–24].

Nearly all PWH are CMV seropositive [25]. Reactivation and asymptomatic CMV shedding occur frequently, particularly at mucosal sites such as the genital or gastrointestinal tract [19, 26, 27]. Men, especially men who have sex with men, tend to have more episodes of genital CMV reactivation than women [14, 28–30], reflecting both biological susceptibility and social determinants of health [19]. Additional risk factors include a history of advanced immunosuppression, suboptimal ART adherence, older age, and coinfection with other common herpesviruses [2, 8, 18].

These epidemiologic overlaps underscore the synergy between CMV and HIV: both disproportionately affect marginalized populations, both persist despite immune pressure, and evidence is growing that together they compound inflammation and accelerate multimorbidity in aging PWH.

## CMV and HIV: A Synergistic Burden

In the pre-ART era, CMV was a leading cause of AIDS-related illnesses, including retinitis, encephalitis, and colitis. While these severe end-organ complications are now rare, even asymptomatic CMV infection can amplify immune dysfunction in PWH [2].

**Fig. 1:**
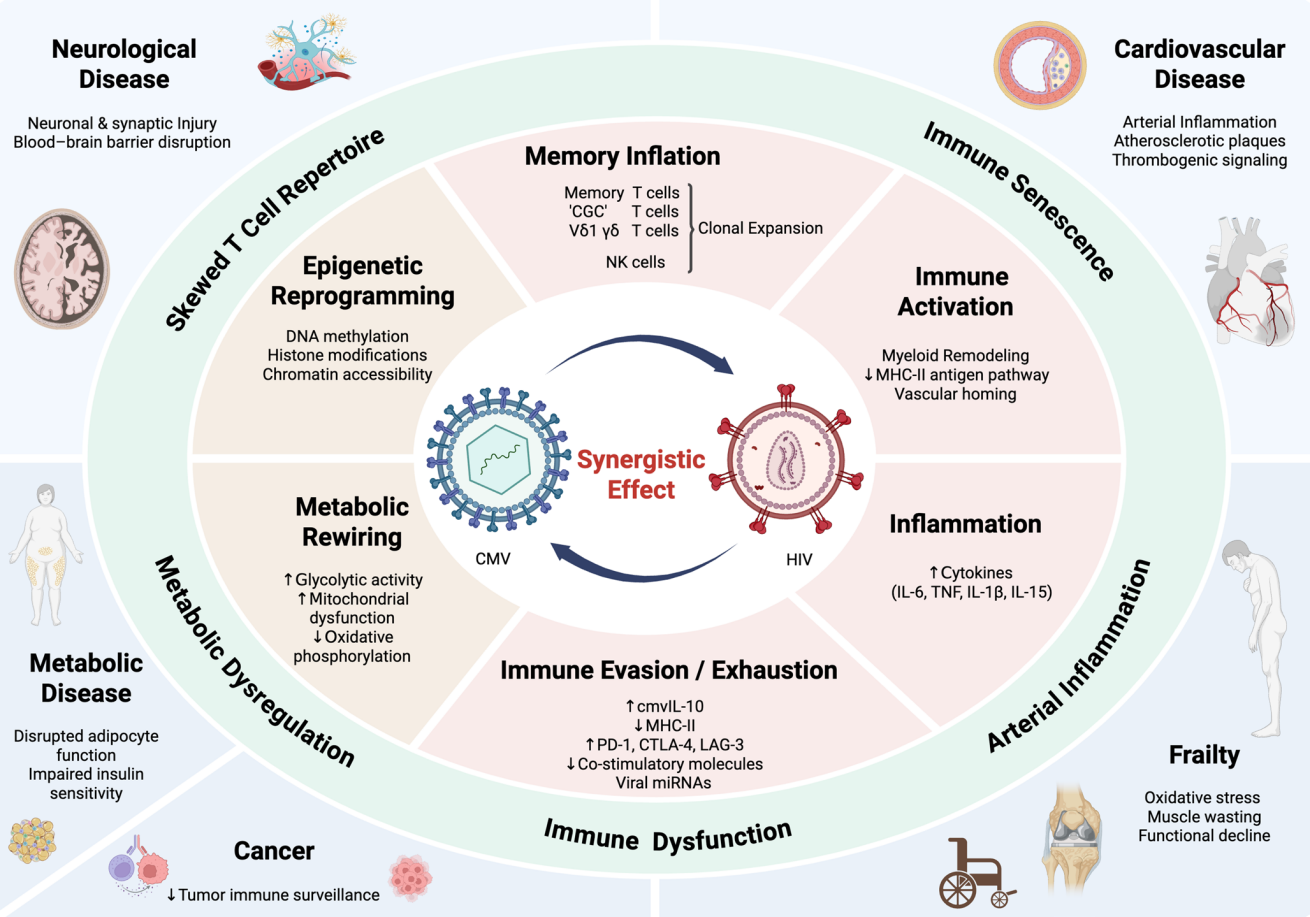
Conceptual Model of HIV–CMV Synergy Driving Immune Dysregulation and Morbidity in PWH. This conceptual model illustrates how persistent HIV and CMV infections act synergistically to drive immune perturbation and contribute to clinical morbidity in people with HIV. The model is structured in four interconnected layers: Layer 1 – Viral Drivers, including HIV and CMV synergistic co-infection, characterized by bidirectional interactions between HIV-related immune dysregulation and CMV-driven immune activation (CMV= cytomegalovirus); Layer 2 – Direct Mechanistic Effects including Memory Inflation, Inflammation and Immune Activation, Immune Evasion/Exhaustion, Metabolic Rewiring; Epigenetic Reprogramming ('CGC'= CX3CR1+– GPR56+ – CD57+, NK= Natural Killer, Vδ1 γδ= Vδ1 γδ T cells, MHC= Major Histocompatibility Complex); Layer 3 – Indirect Biological Consequences, driven by changes in immune cell diversity and function, accelerated immunosenescence, chronic vascular inflammation and other persistent inflammatory processes, and dysregulated metabolic pathways; Layer 4 – Clinical Outcomes, reflecting increased risk and end-organ damage of cardiovascular, metabolic and neurological diseases, frailty and functional decline, and reduced immune surveillance

HIV-related immune dysregulation facilitates CMV reactivation, while CMV-driven immune activation may contribute to HIV persistence [11, 18, 31–33]. Both viruses establish latency, reactivate intermittently, and exploit overlapping reservoirs in the gut, genital tract, and central nervous system [34–38]. In ex vivo studies and animal models, local CMV reactivation disrupts epithelial barriers, promotes microbial translocation, and contributes to immune activation [39]. Importantly, asymptomatic CMV shedding, even without detectable viremia, has been linked to heightened T-cell activation and proliferation [5, 32, 36]. This environment may foster clonal expansion of HIV-infected CD4^+^ T cells, reinforcing the long-lived HIV reservoir [18]. Thus, CMV and HIV may act as “partners in persistence,” each stabilizing the other through chronic or recurrent antigenic stimulation (Fig. 1). CMV-driven immune activation can help maintain the HIV reservoir by promoting clonal proliferation of latently infected CD4⁺ T cells, reshaping trafficking/tissue homing, modulating antiviral immunity (e.g., inflammatory cytokine milieus, T-cell exhaustion), and directly increasing HIV trasncription, among other mechanisms [40–44].

## Impact of CMV on the Immune System: Friend or Foe?

Adaptive T Cells: Inflation and Senescence.From primary infection to lifelong latency and repeated reactivation, CMV shapes the immune system across the lifespan [32]. While this footprint helps maintain viral control, it also fuels chronic inflammation and features of immunosenescence that are amplified in PWH [45].

### Adaptive T Cells: Inflation and Senescence

One hallmark of CMV infection is expansion of effector memory T cells at the expense of naïve and central memory pools [32]. In some individuals, up to 30% of circulating CD8⁺ and CD4⁺ T cells recognize CMV antigens [46].These expanded clones often express CD57 and lack CD28, markers of terminal differentiation and reduced proliferative capacity [46–50]. Functionally, these highly differentiated T cells produce proinflammatory cytokines and display cytotoxic potential, but are less capable of responding to new antigens [46–50]. In PWH, this skewed repertoire compounds HIV-driven immune dysfunction [51]. Expanded CMV-specific T cells frequently express the vascular-homing fractalkine receptor CX3CR1 and the adhesion receptor GPR56, both of which facilitate vascular homing [52]. These ‘CGC’ (*C*X3CR1^+^, *G*PR56^+^, *C*D57^+^) cells have been recovered from atherosclerotic plaques and adipose tissue, linking CMV-driven immunity to cardiometabolic complications, as highlighted in section VI [53–56]. Importantly, CMV-specific CD4⁺ T cells with cytotoxic features can also harbor latent HIV, directly tying CMV to reservoir persistence [43, 57].

### Non-Conventional T Cells: γδ Expansions

CMV also shapes non-classical lymphocyte populations. Chronic infection drives clonal expansion of Vδ1 γδ T cells, which acquire adaptive-like, effector-memory phenotypes with restricted repertoires [58]. These long-lived populations correlate with CMV burden and show evidence of tissue residency [58–60]. In PWH, γδ T-cell expansions have been linked to altered levels of cell-associated HIV RNA, suggesting a complex role that may include both antiviral defense and immune remodeling [61].

### Innate Immunity: NK Cells and Myeloid Remodeling

CMV also reshapes innate immunity. It alters Natural Killer (NK) cell biology, promoting the expansion of long-lived “adaptive” NK cells (CD16^–^, NKG2C^+^) with potent cytotoxicity and memory-like persistence [62–67]. While effective against CMV, their narrowed repertoire reduces flexibility against other pathogens, a trade-off that may impair overall immune resilience in PWH [66]. Latently infected myeloid progenitor cells and monocytes downregulate antigen presentation pathways (e.g., MHC-II) [68] while producing high levels of inflammatory cytokines (e.g. interleukin [IL]-6, TNF, IL-1β, IL-15) [32, 69, 70]. This proinflammatory cascade reinforces immunosenescence and contributes to the chronic inflammatory milieu characteristic of aging with HIV [69].

### Immune Evasion and Exhaustion

CMV persistence depends on sophisticated immune evasion strategies. The virus encodes a homolog of IL-10 (cmvIL-10) that mimics host IL-10, dampening dendritic cell and macrophage activation and antigen presentation, while inducing further host IL-10 production [68, 71–74]. cmvIL-10 binds the human IL-10 receptor with comparable or greater affinity than the endogenous cytokine and exerts stronger suppressive effects on antigen-presenting cells, including inhibition of MHC II and co-stimulatory-molecule expression and reduction of pro-inflammatory cytokine release [72]. This creates a feed-forward loop of immunosuppression. In parallel, chronic CMV stimulation upregulates checkpoint receptors such as PD-1, CTLA-4, LAG-3, and KLRG1, promoting functional exhaustion [47]. CMV also encodes a suite of viral microRNAs that fine-tune host antiviral responses, including those regulating STAT and IRF signaling pathways, further promoting immune evasion [75]. These pathways mirror HIV-driven immune dysfunction, creating synergistic pressure toward premature immune aging. Thus, the ultimate consequences of CMV activity in PWH and its relationship to health are governed by the interplay between pro-inflammatory and anti-inflammatory factors.

### Friend or Foe?

Not all CMV-driven immune alterations are detrimental. In animal models, particularly in younger or otherwise healthy hosts, CMV-induced immune activation has been shown to enhance clearance of bacterial infections and to act as an adjuvant for vaccine responses [76, 77]. Highly differentiated CMV-specific T cells can rapidly respond to reactivation, preventing symptomatic CMV disease, and cross-reactivity may enhance defense against other pathogens [32]. However, in the setting of HIV and aging, these same processes become maladaptive, fueling chronic inflammation, reducing immune diversity, and accelerating comorbidity development [78]. Additional, yet unidentified non-age-related factors may modulate the impact of CMV on the host.

## Cellular Metabolism and Epigenetics

CMV exerts sustained metabolic, transcriptional, and epigenetic pressures on host cells that persist long after primary infection and shape the immune and inflammatory landscape of aging.

### Metabolic Rewiring

During lytic infection, CMV hijacks host metabolism to fulfill the high biosynthetic demands of viral replication, increasing glucose uptake and flux through glycolysis, fatty acid synthesis, and amino acid metabolism [79, 80]. These changes expand nucleotide and lipid pools and support energy-intensive processes required for virion production. Even during latency, infected monocytes and progenitor cells exhibit a distinct metabolic phenotype, characterized by elevated glycolytic activity, mitochondrial dysfunction, and altered oxidative phosphorylation [81, 82]. This metabolic rewiring creates a low-grade proinflammatory environment and may predispose to systemic metabolic disorders, particularly in PWH, who already face ART-related dyslipidemia, insulin resistance, and chronic immune activation [79].

### Epigenetic Imprinting and Trained Immunity

CMV induces lasting epigenetic reprogramming that could perpetuate inflammation, immune aging, and reduced cellular plasticity [22]. Latent CMV infection is associated with host DNA methylation and histone modifications that may bias lymphocytes into long-lived, cytotoxic memory states, changes that could constrain repertoire flexibility [32, 67, 69]. These cellular epigenetic programs are thought to be relatively stable, potentially sustaining expanded pools of CMV-specific T and NK cells and contributing to memory T-cell expansion, a response that might limit reactivation but also accelerate immunosenescence [32, 66, 69]. Related pathways may influence HIV pathogenesis, for example by favoring persistence of infected clones and, in some contexts, biasing toward integration near actively transcribed genomic regions [22, 83].

In addition to epigenetic aging [84], CMV seropositivity has been linked to lower CD4:CD8 ratios and expansion of effector-memory and Terminal Effector Memory cells that re-express the CD45RA antigen (TEMRA) subsets [85]. Large population studies suggest that CMV contributes to inter-individual variation in DNA methylation, with effects that appear to persist after adjusting for immune cell composition [32, 86]. Machine-learning approaches can, in some datasets, predict CMV status from cellular DNA methylation profiles and identify methylation “episcores” that correlate with infection risk, impaired immune differentiation, and dysregulated development [86–88].

### Intersection with HIV and Aging

In summary, CMV-induced metabolic and epigenetic alterations amplify HIV-associated immune dysfunction. Both viruses converge on pathways of mitochondrial injury and metabolic stress: CMV reduces mitochondrial biogenesis and promotes reactive oxygen species production, while HIV infection further depletes mitochondrial DNA and disrupts oxidative phosphorylation [89]. Together, these effects accelerate cellular senescence, telomere erosion, and metabolic exhaustion [34]. Epigenetically, CMV-driven promoter hypermethylation of interferon-stimulated genes, along with altered histone methylation at antiviral loci, can suppress innate antiviral responses, which is another mechanism that favors HIV persistence and reservoir stability [86].

Collectively, CMV reshapes cellular metabolism and reprograms the epigenome, reinforcing the inflammatory and metabolic dysfunctions that underlie HIV-associated multimorbidity [79]. These persistent molecular imprints provide mechanistic insight into how CMV contributes to the premature biological aging observed in PWH.

## CMV-Associated Comorbidities in Aging and HIV

The immune activation, inflammation, and metabolic remodeling driven by CMV increase risk for a spectrum of aging-related diseases [8]. In PWH, persistent CMV replication and immune stimulation are linked to frailty, cardiovascular disease (CVD), brain disorders, metabolic syndrome, and certain cancers, conditions that remain disproportionately prevalent despite suppressive ART [90].

### CVD and CMV in PWH.

CMV increases the risk for atherosclerosis, ischemic heart disease, myocardial infarction, and CVD mortality, though effect sizes vary [8, 52, 91–97]. The association appears stronger in immunosuppressed groups such as PWH on ART and solid organ transplant recipients than in the general population [97]. For example, in solid organ transplant recipients, short-course ganciclovir after heart transplantation halved post-transplant atherosclerosis in one trial, consistent with a CMV-modifiable component of vascular risk [98].

CMV may contribute to CVD via (i) direct vascular infection of endothelial and smooth muscle cells, with viral DNA detected in more than 80% of atherosclerotic plaques in one endarterectomy series [99–101]; (ii) endothelial activation, including extracellular vesicle-mediated antigen transfer and upregulation of cytokines and adhesion molecules that increase permeability and leukocyte recruitment [102–107]; (iii) thrombogenic signaling, including cell-independent thrombin generation [99, 100, 108–110]; and (iv) recruitment and expansion of vascular-homing CX3CR1⁺ T cells and monocytes with cytotoxic/senescent phenotypes that produce IL-6 and TNF-α, amplifying plaque inflammation and instability [97, 111–114].

Higher CMV-specific CD8⁺ T-cell frequencies and CMV-specific IgG levels correlate with atherosclerosis measures in treated PWH [54, 113, 115–117], and CMV seropositivity has been linked to a more than two-fold increased risk of subsequent CVD events [8]. Not all findings are uniform—for example, in a REPRIEVE substudy, CMV IgG titer associated with inflammation but not total plaque burden [91], though sensitivity analyses suggested links with high-risk plaque features [100].

Taken together, mechanistic and epidemiologic evidence implicate CMV as a driver of vascular inflammation, plaque progression, and adverse cardiovascular outcomes. These findings highlight CMV-targeted strategies, antiviral therapy, immunomodulation, or vaccination, as an underexplored but potentially valuable avenue to mitigate cardiovascular risk in PWH.

### CMV and Brain Health

Growing evidence supports that CMV has lasting effects on brain structure and mental health across the lifespan [118]. CMV infection has been linked to deficits in hearing, memory, language, and overall cognitive function, with symptomatic disease and higher antibody levels predicting greater impairment [119].

In older adults with and without HIV, CMV seropositivity has been associated with accelerated cognitive decline and increased risk of Alzheimer’s disease [120, 121]. Even in non-elderly adults without HIV, CMV seropositivity correlates with poorer cognitive performance [122], and in PWH, elevated anti-CMV IgG titers and robust CMV-specific CD4⁺ responses have been linked to slower processing speed, memory deficits, and worse cognition despite suppressive ART [51, 123, 124]. Yet findings are not always consistent [125], highlighting the need for more research to better understand when and how CMV contributes to brain injury.

Beyond cognition, CMV has also been implicated in psychiatric conditions, such as depression, bipolar disorder, schizophrenia, and suicide risk [126–128]. Higher CMV antibody titers are consistently associated with increased risk of these disorders, and contextual factors such as chronic stress, low socioeconomic status, and reduced immune resilience appear to interact with CMV to amplify its psychiatric effects [129]. These findings support that the impact of CMV on brain health extends beyond cognition and is part of a larger web linking infection, stress, and mental health.

Neuroimaging studies provide convergent evidence linking CMV infection to structural brain changes. Multiple studies have shown correlations between CMV antibody titers and amyloid deposition [130], reduced hippocampal and cortical volumes, and accelerated age-related atrophy [131] in people without HIV, effects that may vary by sex [132] and appear stronger in PWH or individuals with preexisting mental health disorders. CMV infection has also been associated with smaller dentate gyrus volume [133] and smaller cortical surface area [134] in people with psychiatric disorders. By contrast, these associations are often absent in otherwise healthy individuals, suggesting that host vulnerability may modulate CMV’s neurotropic effects.

Mechanistically, CMV contributes to these brain health disorders through chronic immune activation, oligoclonal CD8⁺ expansion, immunosenescence, and production of proinflammatory cytokines that disrupt the blood–brain barrier [89, 135] leading to neuronal and synaptic injury [2, 5, 120]. CMV DNA has been detected in brain tissue even in the absence of clinical encephalitis, supporting a role for subclinical reactivation and local immune-mediated damage [123, 136]. In PWH, CMV may further promote HIV replication and reservoir persistence, which have been associated with worse cognitive outcomes [137, 138].

Taken together, these findings position CMV as a potentially modifiable risk factor for brain health disorders. However, inconsistencies across studies and cohorts highlight the need for mechanistic, longitudinal, and interventional work to determine whether antiviral, vaccine, or immunomodulatory strategies can prevent, modify, or alleviate these effects.

### Frailty and Physical Function Declines

CMV infection has been linked to disability [139] and impaired physical function and frailty, a state of heightened vulnerability in older adults without HIV [140]. In the Women’s Health and Aging Study, both CMV seropositivity and higher antibody titers were associated with frailty [141, 142], after accounting for age and comorbid disease burden. CMV reactivation are also higher in frail than non-frail older adults [143]. Similar associations occur in PWH, where higher CMV antibody titers predict frailty or impairment in physical function in middle-aged and older adults [144, 145]. Findings are not entirely consistent, though: some studies have failed to find associations [146, 147].

The immune effects of CMV have again been implicated with frailty. In PWH, breadth and polyfunctionality of CMV-specific T cell responses accelerate immune senescence and are associated with increased risk of frailty [78, 148]. CMV-related pro-inflammatory cytokines such as IL-6 and TNF-α also appear to contribute to muscle wasting and functional decline [149, 150]. Additionally, CMV is linked to oxidative stress and mitochondrial dysfunction, which can impair muscle performance in aging populations [150].

Taken together, these findings support that CMV promotes frailty both directly, through local tissue and muscle damage, and indirectly, through sustained T and B cell responses that drive chronic inflammation and immune aging.

### Metabolic Dysregulation and Adipose Tissue Inflammation

Evidence increasingly supports a role for CMV in metabolic dysregulation, particularly in older or immunosuppressed populations. In the Leiden 85-plus cohort (a population-based cohort of 85-year-old adults living in the community in the Netherlands, without health-based selection criteria), CMV seropositivity was associated with higher glycosylated hemoglobin, elevated non-fasting glucose, and greater prevalence of type 2 diabetes mellitus [151]. Among renal transplant recipients, asymptomatic CMV infection was associated with impaired insulin secretion and increased risk of incident diabetes [152], findings confirmed in a meta-analysis of more than 1,300 kidney transplant patients, which demonstrated nearly a two-fold increased risk of post-transplant diabetes [153]. In younger, non-transplant recipients, findings have been more heterogeneous. In the National Health and Nutrition Examination Survey (NHANES), CMV seropositivity was associated with metabolic syndrome in women but not in men, with effects modified by obesity [154]. Similarly, in the United Kingdom Household Longitudinal Study, CMV seropositivity was associated with higher glycosylated hemoglobin and reduced High-Density Lipoprotein (HDL) cholesterol independent of obesity or metabolic risk factors [155]. These studies support the conclusion that CMV contributes to metabolic dysregulation, though effects vary by age, adiposity, sex, and clinical context.

Mechanistic studies highlight the adipose compartment as a central site of CMV interaction with host metabolism. Adipose tissue is permissive to diverse viral pathogens, including influenza [156], murid herpesviruses (as models for human herpesviruses) [157, 158], HIV [159, 160], SIV [161], and CMV [162, 163]. Analysis of the Genotype Tissue Expression transcriptome atlas identified adipose tissue as one of the richest sources of CMV transcripts among more than 30 tissue types, consistent with chronic, low-level viral activity [163]. In this setting, CMV infection drives persistent antiviral immune responses that disrupt tissue metabolic function.

Experimental evidence strengthens the link between CMV and metabolic dysfunction. In animal models, CMV infection induces adipose tissue inflammation and insulin resistance [164–166]: both mice and non-human primates develop inflamed, cytokine-rich adipose depots after infection [159, 167–170]. In vitro, CMV infection of human adipose-derived stromal/stem cells impairs adipocyte differentiation and alters their immunomodulatory function, indicating direct viral interference with tissue homeostasis [171].

 In vivo studies in PWH support these findings. In subcutaneous and visceral adipose tissue, people with CMV, especially those with diabetes, have increased CGC effector-memory and TEMRA cells with proinflammatory and cytotoxic profiles that recognize CMV antigens [55, 117, 167, 172, 173]. Single-cell transcriptomics and T-cell receptor (TCR) analyses confirm enrichment of CMV-specific clones, particularly in visceral fat, where CMV persistence drives expansion of tissue-resident cytotoxic T cells, cytokine production, and metabolic disturbances such as hyperglycemia [174, 175].

Together, these findings indicate that CMV establishes reservoirs within adipose tissue and elicits chronic antiviral immune responses that disrupt adipocyte function, impair insulin sensitivity, and contribute to systemic metabolic dysregulation in PWH.

### Cancer and Immune Surveillance

CMV exerts dual and context-dependent roles in cancer biology. On one hand, chronic CMV infection impairs immune surveillance, promoting an inflammatory and immunosuppressive environment that may reduce tumor control [32, 34, 176]. CMV seropositivity increases the risk of certain solid tumors and lymphomas, particularly in immunocompromised populations [177, 178]. CMV antigens and DNA are present in several tumor types, including glioblastoma and colorectal cancer, suggesting either direct viral involvement or bystander effects within the tumor niche, but this is still controversial [178]. Conversely, CMV-derived antigens have been harnessed as targets in experimental oncolytic and vaccine-based immunotherapies to boost anti-tumor immunity [179]. In the context of HIV, however, the chronic inflammation, T-cell exhaustion, and reduced NK-cell diversity induced by CMV impairs tumor immune surveillance and amplifies cancer risk.

## Therapeutic Strategies Targeting CMV

Recognition of CMV as a modifiable driver of inflammation, immune dysfunction, and aging has sparked growing interest in strategies that target CMV or its downstream pathways [180, 181]. To date, no interventions have been approved for CMV suppression in asymptomatic immunocompetent or ART-suppressed PWH; however, emerging antiviral, immunomodulatory, and vaccine-based approaches are beginning to redefine this therapeutic landscape.

### Antiviral therapies

Classical CMV antivirals such as ganciclovir, valganciclovir, cidofovir, and foscarnet are effective for clinical disease but limited by toxicity and resistance, making them unsuitable for long-term prevention in otherwise healthy PWH. Newer agents, particularly letermovir, a terminase inhibitor approved for CMV prophylaxis in transplant recipients [182], offer a more favorable safety profile, with no myelotoxicity or renal dosing concerns, although it inhibits CYP3A4 which can cause drug–drug interactions. Letermovir is inactive against other herpesviruses but has efficacy in suppressing CMV reactivation across several settings [183–185]. Studies support that letermovir may restore tissue integrity and reduce inflammation in people with CMV [39, 186]. Resistance remains rare, although delayed recovery of CMV-specific T-cell responses has been reported [187]. Preliminary results from ACTG A5383, a randomized, open-label, phase II clinical trial evaluating 48 weeks of letermovir in PWH on ART (NCT04840199) include a reduction in cytokine production (IL-1β, IL-6R), an increase in CD4/CD8 ratio, and improvements in physical function [188], particularly in participants with lower CD4^+^ T cells and in women. These preliminary findings are highly relevant for guiding future trials in aging populations.

### Vaccines

Despite decades of effort, no CMV vaccine has been licensed [189]. Several platforms, including recombinant glycoprotein B, vector-based candidates, and mRNA vaccines, have shown immunogenicity and partial efficacy in clinical trials [190–192]. In PWH, a successful vaccine could reduce reactivation and systemic inflammation, but the durability of protection and efficacy in immunocompromised hosts remain uncertain. There is an ongoing clinical trial in the ACTG (A5355; NCT05099965) to study the safety and immunogenicity of Triplex, a modified vaccinia Ankara viral vector vaccine targeting CMV, in PWH on ART, with initial results expected in 2026. The resurgence of CMV vaccine development in the mRNA era may accelerate progress.

## Knowledge Gaps and Future Directions

Despite compelling evidence linking CMV to immune aging and comorbidity in PWH, many critical questions remain unanswered (Table 1).

**Table 1:** Scientific gaps and future directions

Domain	Scientific Gaps	Research Need	Impact/translation
Causality	Unclear if CMV is a driver vs. bystander in aging-related outcomes.	Randomized CMV suppression/vaccine trials with aging and comorbidity endpoints	Define CMV as a modifiable driver of disease, enabling intervention-based prevention strategies
Tissue-specific mechanisms	CMV contribution to specific diseases (CVD, cognition, frailty, diabetes) not fully defined	Spatial, single-cell, multi-omic mapping of CMV reservoirs and immune responses in tissues	Identify key tissues and pathways for intervention
Host and environmental modifiers	Impact of age, sex, obesity, ART, or stress and other vulnerability factors not well defined	Inclusion of diverse, representative populations and stratified analyses	Enable precision risk stratification and tailored interventions
Biomarkers	No reliable marker of CMV activity, burden, or pathogenic relevance	Validation of episcores, TCR signatures, CMV shedding patterns, and tissue-based markers	Enable causal inference, patient stratification, and treatment monitoring
Therapeutics	Limited availability of safe, low-toxicity, low-cost CMV therapies	Development of scalable antivirals and vaccines with equitable access	Reduce morbidity and mortality in all people with HIV, not just high-risk subgroups
Integration with HIV cure & aging trials	CMV rarely considered in HIV cure or aging trials	Routine inclusion of CMV endpoints and biomarkers in HIV trials	Enhance interpretability of immune, inflammatory and aging outcome

CMV= Cytomegalovirus; CVD= Cardiovascular disease; ART = Antiretroviral treatment; TCR = T-cell receptor; PWH= People with HIV

### Defining Causality

Most data are observational [150, 193, 194], leaving uncertainty about whether CMV is a driver or a bystander in aging-related outcomes. Randomized trials testing antivirals (e.g., letermovir) or vaccines with endpoints related to biological aging, inflammation, and comorbidity are urgently needed to determine causality.

### Mechanistic Pathways

While the effects of CMV on T-cell expansion and inflammation are well described, the precise mechanisms linking CMV to specific diseases, such as CVD, neurocognitive decline, frailty, and diabetes, remain incompletely understood. Priorities include mapping tissue reservoirs (e.g., adipose, vasculature, brain), clarifying the relative roles of viral reactivation versus latent imprinting, and integrating single-cell, spatial, and multi-omic approaches to resolve cell-type and tissue-specific pathways.

### Host and Environmental Modifiers

Not all individuals with CMV infection develop the same degree of immune remodeling or comorbidity. Factors such as sex, obesity, co-infections (e.g., HIV, Hepatitis B virus, Epstein-Barr virus), ART regimen, genetics, and psychosocial stress likely influence outcomes. Understanding these modifiers could inform precision risk stratification and identify vulnerable subgroups most likely to benefit from intervention.

### Biomarkers and Predictors

Current measures such as CMV IgG titers are crude proxies of viral burden [142]. Emerging biomarkers, including epigenetic “episcores,” CMV-specific TCR repertoires, DNA methylation patterns, and tissue-based measures of viral activity, may provide more precise indicators of disease risk and response to therapy. However, assessing CMV shedding requires collection of mucosal secretions or tissues, which can be logistically challenging. Validation and standardization of these biomarkers will be essential for clinical trials and individualized monitoring.

### Therapeutic Development

Existing antivirals are limited by toxicity [183, 195, 196], and new agents, vaccines, and host-directed therapies (e.g., IL-10 blockade, checkpoint inhibitors, senolytics, adoptive cell transfer) remain underexplored in PWH. Carefully designed interventional studies should prioritize safety, durability, and effects on multimorbidity rather than virologic suppression alone.

### Integration with HIV Cure and Aging Research

CMV profoundly shapes immune function, reservoir persistence, and inflammaging [32]. Future HIV cure trials and aging studies should incorporate CMV status, activity, and interventions as key covariates, recognizing CMV as a major environmental determinant of outcomes.

## Conclusion

CMV infection is nearly universal among PWH and has far-reaching effects on immunity, metabolism, and tissue health [26]. By driving chronic immune activation, clonal T-cell expansions, and persistent epigenetic and metabolic remodeling, CMV contributes to accelerated aging and a broad spectrum of comorbidities, including cardiovascular disease, cognitive decline, frailty, cancer, and metabolic dysfunction.

Although much of the current evidence is observational, converging data from human cohorts, tissue studies, and experimental models support CMV as a central driver of inflammaging and multimorbidity in HIV [18]. Importantly, CMV represents a modifiable risk factor. Emerging antivirals, vaccines, and host-directed interventions provide new opportunities to test whether CMV suppression can improve healthspan in PWH.

In contrast to broad host immunomodulation, which has shown non-trivial infectious risks in other contexts, CMV-directed strategies may represent a more focused and potentially safer way to attenuate inflammation and vascular risk in treated PWH. Integrating CMV assessment and targeted interventions into HIV cure and aging research could yield transformative insights and open novel paths to comorbidity prevention. Taken together, these findings position CMV not as a silent bystander but as a therapeutic target whose modification may extend both lifespan and healthspan in people aging with HIV.

Collectively, these approaches reflect a paradigm shift: treating CMV not only as a pathogen but as a modifiable determinant of multimorbidity and biological aging. The convergence of antiviral efficacy, multi-omic biomarker improvement, and epidemiologic links to morbidity provides a compelling rationale for interventional studies. Given that infection-related cancers and inflammation-driven comorbidities are now among the leading causes of death in ART-suppressed PWH, and the plausibility that CMV may contribute to these risks, advancing clinical trials of CMV interventions is crucial. 

## Key References

- Freeman ML, Lederman MM, Gianella S. Partners in Crime: The Role of CMV in Immune Dysregulation and Clinical Outcome During HIV Infection. Curr HIV/AIDS Rep. 2016;13(1):10–9.
  - ○ The review highlights how persistent CMV contributes to chronic immune activation and inflammation in PWH on antiretroviral therapy, potentially accelerating age-related diseases such as cardiovascular and neurocognitive disorders.
- Christensen-Quick A, Vanpouille C, Lisco A, Gianella S. Cytomegalovirus and HIV Persistence: Pouring Gas on the Fire. AIDS Res Hum Retroviruses. 2017;33(S1):S-23-S-30.
  - ○ This review explores how CMV co-infection may promote HIV persistence despite antiretroviral therapy by driving chronic immune activation and modulating cellular pathways that sustain the latent HIV reservoir, thereby hindering its eradication.
- Müller L, Di Benedetto S. Immunosenescence and Cytomegalovirus: Exploring Their Connection in the Context of Aging, Health, and Disease. Int J Mol Sci. 2024;25(2):753.
  - ○ This review examines the complex interaction between immunosenescence and CMV, highlighting how chronic CMV infection shapes the immune system, alters inflammatory profiles, and contributes to age-related diseases.
- Powers C, De Filippis V, Malouli D, Fruh K. Cytomegalovirus immune evasion. Curr Top Microbiol Immunol. 2008;325:333–59.
  - ○ The review highlights the wide range of immune evasion mechanisms employed by CMV, targeting both innate and adaptive immunity, making it a key model for understanding host–pathogen interactions.
- Hmiel L, Zhang S, Obare LM, Santana MADO, Wanjalla CN, Titanji BK, et al. Inflammatory and Immune Mechanisms for Atherosclerotic Cardiovascular Disease in HIV. Int J Mol Sci. 2024;25(13):7266.
  - ○ This review summarizes current knowledge on immune and inflammatory mechanisms contributing to atherosclerotic cardiovascular disease in PWH, emphasizing the roles of viral products, cytokines, immune cell dysregulation, and co-infections, as well as potential therapeutic targets to reduce cardiovascular risk.
- Zhu W, Liu S. The role of human cytomegalovirus in atherosclerosis: a systematic review. Acta Biochim Biophys Sin. 2020;52(4):339–53.
  - ○ This systematic review analyzes the association between CMV infection and atherosclerosis, highlighting how CMV may influence vascular and immune cells through mechanisms such as oxidative and endoplasmic reticulum stress, autophagy, lipid metabolism, and miRNA regulation, thereby contributing to vascular injury and cardiovascular disease.
- Bergstedt J, Azzou SAK, Tsuo K, Jaquaniello A, Urrutia A, Rotival M, et al. The immune factors driving DNA methylation variation in human blood. Nat Commun. 2022;13(1):5895.
  - ○ This study identifies key immune and lifestyle factors influencing DNA methylation variation in human blood and reveals how latent CMV infection significantly shapes the methylome, contributing to age-related epigenetic changes and emphasizing the strong impact of cellular composition and genetic variation on epigenetic regulation.
- Gale SD, Farrer TJ, Erbstoesser R, MacLean S, Hedges DW. Human Cytomegalovirus Infection and Neurocognitive and Neuropsychiatric Health. Pathogens. 2024;13(5):417.
  - ○ This review examines the links between CMV infection and neurocognitive as well as neuropsychiatric disorders, summarizing evidence of associations between CMV and cognitive decline, dementia, and mood or developmental disorders, while highlighting the inconsistency of current findings and the need for longitudinal and therapeutic studies to clarify these relationships.
- Kirkham FA, Shwe PS, Mensah E, Rajkumar C. The relationship of cytomegalovirus with physical functioning and health-related quality of life in older adults. Eur Geriatr Med. 2025.
  - ○ This paper show how CMV seropositivity is significantly linked to lower physical functioning and quality-of-life scores in older adult, although its direct role in sarcopenia remains unclear.
- Erlandson KM, Allshouse AA, Rapaport E, Palmer BE, Wilson CC, Weinberg A, et al. Physical Function Impairment of Older, HIV-Infected Adults Is Associated with Cytomegalovirus Immunoglobulin Response. AIDS Res Hum Retroviruses. 2015;31(9):905–12.
  - ○ This case–control study demonstrates that higher CMV-specific IgG levels are strongly associated with impaired physical function in older adults with well-controlled HIV infection, suggesting that systemic inflammation and immune suppression may mediate this relationship.
- Gianella S, Erlandson K, Kitch D, Qiu S, Fukazawa Y, Meneses M. Letermovir for CMV Suppression Improves Immunologic and Functional Aging. Abstract 182, CROI conference, March 9–12, 2025; San Francisco, CA.
  - ○This randomized clinical trial in PWH and CMV coinfection found that 48 weeks of letermovir, a CMV-specific terminase inhibitor, led to sustained reductions in inflammatory markers, improved CD4/CD8 ratios, and enhanced physical function, suggesting that CMV suppression could counteract immunologic and functional aging.

## Data Availability

No datasets were generated or analysed during the current study.
